# Efficient Rank-Based Diffusion Process with Assured Convergence

**DOI:** 10.3390/jimaging7030049

**Published:** 2021-03-08

**Authors:** Daniel Carlos Guimarães Pedronette, Lucas Pascotti Valem, Longin Jan Latecki

**Affiliations:** 1 Department of Statistics, Applied Mathematics and Computing (DEMAC), São Paulo State University (UNESP), Rio Claro 13506-900, Brazil; lucas.valem@unesp.br; 2 Department of Computer and Information Sciences, Temple University, Philadelphia, PA 19122-1801, USA; latecki@temple.edu

**Keywords:** diffusion, rank, image retrieval, convergence

## Abstract

Visual features and representation learning strategies experienced huge advances in the previous decade, mainly supported by deep learning approaches. However, retrieval tasks are still performed mainly based on traditional pairwise dissimilarity measures, while the learned representations lie on high dimensional manifolds. With the aim of going beyond pairwise analysis, post-processing methods have been proposed to replace pairwise measures by globally defined measures, capable of analyzing collections in terms of the underlying data manifold. The most representative approaches are diffusion and ranked-based methods. While the diffusion approaches can be computationally expensive, the rank-based methods lack theoretical background. In this paper, we propose an efficient Rank-based Diffusion Process which combines both approaches and avoids the drawbacks of each one. The obtained method is capable of efficiently approximating a diffusion process by exploiting rank-based information, while assuring its convergence. The algorithm exhibits very low asymptotic complexity and can be computed regionally, being suitable to outside of dataset queries. An experimental evaluation conducted for image retrieval and person re-ID tasks on diverse datasets demonstrates the effectiveness of the proposed approach with results comparable to the state-of-the-art.

## 1. Introduction

For decades, the evolution of image retrieval approaches was mainly supported by the development of novel features for representing the visual content. Other relevant stages of the retrieval pipeline were often neglected [1]. Even in the era of deep learning-based features, retrieval systems often perform comparisons by computing measures which consider only pairs of images and ignore the relevant information encoded in the relationships among images. Traditionally, such measures are defined based on pairwise dissimilarities between features represented in a high dimensional Euclidean space [2].

To go beyond pairwise analysis, post-processing methods have been proposed with the aim of increasing the effectiveness retrieval tasks without the need for user intervention [3,4,5,6]. Such unsupervised methods aim at replacing similarities between pairs of images by globally defined measures, capable of analyzing collections in terms of the underlying data manifold, i.e., in the context of other objects [2,4].

Diversified context-sensitive methods have been exploited by post-processing approaches for retrieval tasks. Among them, two categories can be highlighted as very representative of existing methods: *diffusion processes* [3,7,8] and *rank-based* approaches [6,9,10]. The most common diffusion processes are inspired by random walks [5], and therefore supported by a strong mathematical background. Very significant improvements to retrieval performance have been achieved by such methods [3,7,8]. However, they often require high computational efforts, mainly due to the asymptotic complexity associated with matrices multiplication or inversion procedures.

More recently, rank-based methods also have attracted a lot of attention, mainly due to relevant similarity information encoded in the ranked lists [11,12]. While the rank-based strategies also achieve very significant effectiveness gains, such methods lack a theoretical basis and convergence aspects are mainly based on empirical analysis [13]. On the other hand, a positive aspect is related to the low computational costs required. Most of relevant similarity information is located at top rank positions, reducing the amount of data which needs to be processed and enabling the development of efficient algorithms [14]. In fact, efficiency aspects assumed a relevant role last years for both diffusion and rank-based methods, especially regarding its application to query images outside of the dataset [15,16].

**Figure 1:**
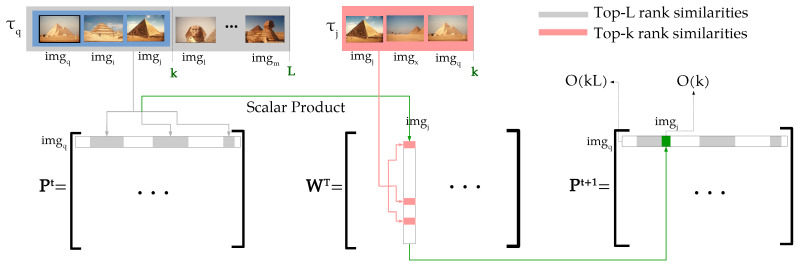
Our efficient algorithm constrained to top-*L* rank positions exploited for efficiently computing the Rank Diffusion.

In this paper, we propose a diffusion process completely defined in terms of ranking information. The method is capable of approximating a diffusion process based only on the top positions of ranked lists, while assures its convergence. Therefore, since it combines diffusion and rank-based approaches, both efficiency and theoretical requirements are met. The main contributions of this work are three-fold and can be summarized as follows:

- *Efficiency and Complexity Aspects:* traditionally, diffusion processes compute the elements of affinity matrices through successive multiplications, considering all the collection images. To reduce the computational costs, sparse affinity matrices [8] are employed for the first iteration. However, there is no guarantees that the multiplied matrices are also sparse and classic diffusion methods do not know in advance where the non-zero values appear in the matrices computed over the iterations. Therefore, a full multiplication of $O(n^{3})$ time complexity is required for each iteration. In opposite, the proposed method keeps the sparsity of matrices by computing only a small subset of affinities, which are indexed through rank information. First, the proposed method derives a novel similarity measure based only on top-*k* ranking information. Then, only the matrix positions indexed by the top-*L* rank positions (L>k) are computed. Inspired by [9,17], the overlap between ranked lists and, equivalently, between elements of rows/columns being multiplied is considered. Figure 1 illustrates this process for computing a matrix row, with sparsity depicted in white. The operation is constrained to top-*L* rank positions with time complexity of O(kL), and therefore O(1) for each row (with *L* constant), and hence O(n) for all rows, i.e., the whole dataset. Therefore, the method computes only a small subset of operations required by diffusion processes, reducing the conventional time complexity for the whole dataset from $O(n^{3})$ to O(n).
- *Theoretical Aspects:* while convergence is an aspect well-defined and widely studied for diffusion processes [5,18], the same cannot be said about rank-based approaches. In fact, the use of classic proofs for rank-based approaches is not straight-forward and the convergence is still an open topic when considering rank information [19], only analyzed through empirical studies [13]. Once the connection between diffusion and rank-based methods is formally established, we discuss the extension of convergence properties from diffusion to the proposed rank-based approach. To the best of our knowledge, this is the first work which presents a formal proof of convergence of rank-based methods.
- *Unseen Query Images:* most of both diffusion and ranked-based methods, consider the query image to be contained in the dataset. Alternatively, a query image can be included in the dataset at query time. However, even an efficient algorithm of O(n) is unfeasible to be executed on-line for larger datasets. A more recent research trend consists on post-processing approaches for efficiently dealing with unseen queries at query time [15,16]. The proposed method also allows its use for unseen queries through a simple regional diffusion constrained to top-*L* rank positions of the query, which can be computed in O(1).

An extensive experimental evaluation was conducted considering different retrieval scenarios. The evaluation for general image retrieval tasks was conducted on 6 public datasets and several features, including global (shape, color, and texture), local, and convolution-neural-network-based features. The performance on Person reidentification (re-ID) tasks was also evaluated on two recent datasets using diverse features. The proposed method achieves very significant gains, reaching up to +40% of relative gain on image retrieval and +50% on person re-ID tasks. Comparisons with various recent methods on different datasets were also conducted and the proposed algorithm yields comparable or superior performance to state-of-the-art approaches.

The remainder of this paper is organized as follows. Section 2 discusses the relationship between diffusion, rank-based and the proposed approach. Section 3 formally defines the proposed method, while Section 4 discusses complexity aspects and presents an efficient algorithmic solution. Section 5 describes the conducted experimental evaluation and, finally, Section 6 discusses the conclusions.

## 2. Diffusion, Rank-Based, and the Proposed Method

Diffusion is one of the most widely spread processes in science [17,20]. In the retrieval domain, diffusion approaches rely on the definition of a global measure, which describes the relationship between pairs of points in terms of their connectivity [2,3,4]. In general, diffusion methods start from an affinity matrix, which establishes a similarity relationship among different dataset elements [5].

Let C=$\{y_{1},$$y_{2},$$\cdots ,y_{n}\}$ be an image collection of size *n* = |C|. Let $x_{i}$ denotes a *d*-dimensional representation of an image $y_{i}$ given by a feature extraction function, such that $x_{i}\in R^{d}$. The dataset can be represented by a set X=$\{x_{1},$$x_{2},$$\ldots ,x_{n}\}$ and the affinity matrix W among the elements is often computed by a Gaussian kernel as

(1) $$w_{ij}=\exp\left(-\frac{\rho {\left(x_{i},x_{j}\right)}^{2}}{2\sigma^{2}}\right),$$

where σ is a parameter to be defined and the function ρ is commonly defined by the Euclidean distance $\rho \left(x_{i},x_{j}\right)=\left|\right|x_{i}-x_{j}\left|\right|$.

Most of the diffusion processes [5] are mainly defined in terms of successive multiplications of affinity matrices, such that their high computational cost is mainly caused by the matrix multiplication operations. This is due to the fact that similarities to all images need to be computed, even those with very low similarity values, whose impact on retrieval results is very small. Additionally, even when sparse affinity matrices are considered, such sparsity is not guaranteed to be kept through the iterative matrices multiplications.

While the diffusion methods use the similarity measure computed based on visual features, the rank-based approaches exploit the similarity encoded in ranking information. The rank information is initially represented in terms of ranked lists, which are computed based on the distance function ρ. One key advantage of rank-based models is due to the fact that top positions of ranked lists are expected to contain the most similar images to the query. Therefore, instead of full ranked lists which can be very time-consuming to compute according to the size of the dataset, only a subset (of a fixed size *L*) needs to be considered.

Formally, let $\tau_{q}$ be a ranked list computed in response to a query image $y_{q}$. Let $C_{L}$ be a subset of the collection C, such that $C_{L}\subset C$ and $|C_{L}|=L$. The ranked list $\tau_{q}$ can be defined as a bijection from the set $C_{L}$ onto the set [L]={1,2,⋯,L}. The notation $\tau_{q}\left(i\right)$ denotes the position (or rank) of image $y_{i}$ in the ranked list $\tau_{q}$ according to the distance ρ. Hence, if the image $y_{i}$ is ranked before $y_{j}$ in the ranked list $\tau_{q}$, i.e., $\tau_{q}\left(i\right)<\tau_{q}\left(j\right)$, then ρ($x_{q}$, $x_{i}$) ≤ρ($x_{q}$, $x_{j}$).

In general, to compute a more global similarity measure between two images $y_{i}$ and $y_{j}$, the rank-based methods exploit the similarity between their respective ranked lists $\tau_{i}$ and $\tau_{j}$. Several distinct approaches have been proposed in order to model the rank similarity information. The asymmetry of the *k*-neighborhood sets were exploited in various works [10,21,22]. Rank correlation measures and similarity between neighborhood sets have also been successfully employed [6,23]. More recently, graph [12,24,25] and hypergraph [26] formulations have been used.

In terms of objectives and outputs, both approaches, diffusion and rank-based, are comparable in that both aim at obtaining more global similarity measures that are expected to produce more effective retrieval results. However, each category presents distinct advantages. While rank-based approaches focus on the similarity encoded in the top positions of ranked lists, reducing the computational cost, the diffusion approaches benefit from a strong mathematical background.

In this scenario, the Rank Diffusion Process with Assured Convergence (RDPAC) is proposed in this paper based on an efficient formulation capable of avoiding the computation of small and irrelevant similarity values. The main idea consists of exploiting the rank information to identify and index the high similarity values in the transition and affinity matrices. In this way, the method admits an efficient algorithmic solution capable of computing an effective approximation of diffusion processes. Mostly related to [17], the proposed approach presents relevant novelties: a theoretical convergence analysis, a novel rank similarity measure, a post-diffusion reciprocal step and its capacity of dealing with unseen queries in on-line time. The proof of convergence of the method presented in this work is a topic which has not been addressed for other rank-based approaches. The proposed similarity considers only ranking information. This makes it robust to feature variations, and therefore, suitable for fusion tasks.

## 3. Rank Diffusion Process with Assured Convergence

The presentation of our method is organized in four main steps: (*i*) a similarity measure is defined based on ranking information; (*ii*) a normalization is conducted for improving the symmetry of ranking references; (*iii*) the rank diffusion process is performed, requiring a small number of iterations; (*iv*) a post-diffusion step is conducted for exploiting the reciprocal rank information. Each step is discussed and formally defined in the next sections in terms of matrix operations. The efficient algorithmic solutions are discussed in Section 4.

### 3.1. Rank Similarity Measure

In this work, a novel approach is proposed for defining the affinity matrix W by using a rank-based strategy. Although we mentioned a common retrieval pipeline based on the Euclidean distance, the method requires only the ranked lists, such that any distance measure can be used. Based only on rank information, our approach defines a very sparse matrix and, at same time, allows predicting information about its sparsity. By exploiting the information about sparsity, it is possible to derive efficient algorithmic solutions (discussed in Section 4).

Taking every image in the collection as a query, a set of ranked lists T = $\{\tau_{1},\tau_{2},$⋯,$\tau_{n}\}$ can be obtained. Based on similarity information encoded on the set T, a rank similarity measure is defined. The confidence of similarity information reaches its maximum at top positions and decreases at increasing depths of ranked lists. Hence, a rank similarity measure is proposed by assigning weights to positions inspired by the Rank-Biased Overlap (RBO) [27]. The RBO measure is based on a probabilistic model which considers the probability of a hypothetical user of keeping examining subsequent levels of the ranked list.

The affinity matrix can be computed according to the different sizes of ranked lists. Let *s* denotes the size of ranked lists, the subscript notation $W_{s}$ is used to refer to affinity matrix computed by considering the size *s*. Each position of the matrix $W_{s}$ is defined as follows:

(2) $$w_{s_{ij}}=\left\{\begin{array}{cc} p^{\tau_{i}\left(j\right)} & \mathrm{if}\;\tau_{i}\left(j\right)\leq s\\ 0, & \mathrm{otherwise}, \end{array}\right.$$

where *p* denotes a probability parameter.

In our method, the ranked list size *s* assumes two different values according to the step being executed. During the rank diffusion, the size is defined as s=k, where *k* denotes the number of nearest neighbors (including the point itself). For the normalization, the size is defined as s=L, defining a more comprehensive collection subset, although much smaller than *n*, i.e., k<L≪n.

In this way, both matrices $W_{k}$ and $W_{L}$ are very sparse, which allows an efficient algorithmic approximation of the diffusion process. Beyond a novel formulation for the similarity measure, the rank information is exploited to identify high similarities positions in sparse matrices. By computing only such positions, a low complexity can be kept for the algorithm, which is one of key characteristics of the proposed approach, discussed in Section 4.

### 3.2. Pre-Diffusion Rank Normalization

In contrast to most distance/similarity pairwise measures, the rank measures are not symmetric. Even if an image $y_{i}$ is at top positions of a ranked list $\tau_{j}$, there is no guarantee that $y_{j}$ is well ranked in $\tau_{i}$. As a result, different values are assigned to symmetric matrix elements, such that $w_{s_{ij}}\neq w_{s_{ij}}$, which can negatively affect the retrieval results. In fact, the benefits of improving the symmetry of the *k*-neighborhood relationship are remarkable in image retrieval applications [28].

Therefore, a pre-processing step based on reciprocal ranking information is conducted before the rank diffusion process. The reciprocal rank references have been exploited by other works, usually considering the information of rank position. In our approach the rank similarity measure described in the previous section is used.

The affinity matrix is computed by considering an intermediary size of ranked lists s=L. By slightly abusing the notation, from now on $W_{L}$ denotes a symmetric version of $W_{L}$, i.e., we have

(3) $$W_{L}=W_{L}+W_{L}^{T}.$$

The number of non-zero entries per row in the so normalized matrix $W_{L}$ is defined in the interval [L,2L], depending of the size of intersection among references and reciprocal rank references. Based on the normalized matrix $W_{L}$, the ranking information is updated through a re-sorting procedure. The ranked lists are re-sorted in descending order of affinity score, according to a stable sorting algorithm. The resultant normalized set of ranked lists T is used for the diffusion process, i.e., Equation (2), and consequently, $W_{k}$ used in next section is computed based on T.

We further column-wise normalize matrix $W_{k}$ to a matrix

(4) $$w_{ij}^{\left(t\right)}=\frac{w_{ij}^{\left(t\right)}}{\epsilon +\sum_{c=1}^{n}w_{jc}^{\left(t\right)}},$$

where ϵ is a small constant to ensure that the sum of each column <1.

### 3.3. Rank Diffusion with Assured Convergence

To make the graph diffusion process independent from the number of iterations, accumulation of similarity values over iterations is widely used [29]. For each iteration, the similarity information is diffused through a transition matrix P and added to the similarity information diffused in previous steps. We initialize the transition matrix P as $P^{\left(1\right)}=W_{k}$ and define the iterative diffusion as

(5) $$P^{\left(t+1\right)}=\alpha P^{\left(t\right)}W_{k}^{T}+\left(1-\alpha \right)I,$$

where α is a parameter in the interval (0,1) and I is the identity matrix. The accumulation of similarity values is achieved through the addition of the identity matrix as we will see in the next section. The addition of the identity matrix also contributes to convergence of the iterative process in (5).

Given the asymmetry of the affinity matrix $W_{k}$, due to column-wise normalization in (4), its transposition is used for considering the multiplication among corresponding rank similarity scores. A non-transposed matrix defines reciprocal rank relationships, which is performed as a post-diffusion step, as discussed in Section 3.5.

### 3.4. Proof of Convergence

To prove the convergence of the iterative diffusion process in Equation (5), we first consider its simpler variant defined as

(6) $$\begin{array}{cc} P^{\left(t+1\right)} & =P^{\left(t\right)}\;W_{k}^{T}+I, \end{array}$$

where $P^{\left(1\right)}=W_{k}$.

As we show now, so defined $P^{\left(t\right)}$ is guaranteed to converge. We can transform (6) to

(7) $$\begin{array}{cc} P^{\left(t+1\right)} & =P^{\left(t\right)}W_{k}^{T}+I \end{array}$$

(8) $$\begin{array}{cc} \; & =\left(P^{\left(t-1\right)}\;W_{k}^{T}+I\right)W_{k}^{T}+I \end{array}$$

(9) $$\begin{array}{cc} \; & =P^{\left(t-1\right)}\;{\left(W_{k}^{T}\right)}^{2}+I\;W_{k}^{T}+I \end{array}$$

(10) $$\begin{array}{cc} \; & =W_{k}\;{\left(W_{k}^{T}\right)}^{t}+I\;{\left(W_{k}^{T}\right)}^{t-1}+\ldots +I \end{array}$$

(11) $$\begin{array}{cc} \; & =W_{k}\;{\left(W_{k}^{T}\right)}^{t}+\sum_{i=0}^{t-1}{\left(W_{k}^{T}\right)}^{i} \end{array}$$

Due to the column-wise normalization of the matrix $W_{k}$, the sum of each row of $W_{k}^{T}<1$. This implies $\lim_{t\to \infty }W_{k}\;{\left(W_{k}^{T}\right)}^{t}=0$, and consequently,

(12) $$\begin{array}{cc} \lim_{t\to \infty }P^{\left(t+1\right)} & =\lim_{t\to \infty }\sum_{i=0}^{t-1}{\left(W_{k}^{T}\right)}^{i}={\left(I-W_{k}^{T}\right)}^{-1} \end{array}$$

This proves the convergence of (6) after a sufficient number of iterations. The convergence proof also applies to the diffusion process in Equation (5). We do not use the closed form solution (12) in our experiments, since the matrix inversion is computationally expensive. Both iterative processes accumulate diffused similarity values, and can be viewed as special instances of

(13) $$A^{\left(t\right)}=\sum_{i=0}^{t}A^{i},$$

where A is a graph affinity matrix. Under the assumption that the sum of each row of A<1, which implies that the spectral radius of A is smaller than one, (13) converges to a fixed and nontrivial solution given by $\lim_{t\to \infty }A^{\left(t\right)}={\left(I-A\right)}^{-1}$, which makes independent of the number of iterations.

In contrast, the rank-based diffusion in [17] represents the simplest realization of a diffusion process on a graph as it only computes powers of the graph matrix, i.e., the edge weights at time (or iteration) *t* are given by $A^{t}$. Hence, this process is sensitive to the number of iterations [29]. For example, if the sum of each row of A is smaller than one, then it converges to the zero matrix, in which case determining a right stopping time *t* is critical. To avoid the convergence to zero matrix, a small value of *t* is used as t=k.

### 3.5. Post-Diffusion Reciprocal Analysis

Despite of the relevant similarity information encoded in the reciprocal rank references, such information is not considered during the rank diffusion process. More specifically, for two images $y_{i}$ and $y_{j}$, the diffusion step considers the information encoded in the rank similarity of $y_{i}$ and $y_{j}$ to another images contained in a shared *k*-neighborhood. The information about the rank similarity of these images to $y_{i}$ and $y_{j}$ is not exploited.

Let θ be the final number of iterations used in (5). To aggregate the reciprocal analysis over the gains already obtained by the diffusion process, a post-diffusion step is proposed. The result of the rank diffusion process given by matrix $P^{\left(\theta \right)}$ is subsequently column normalized according to Equation (4). The post-diffusion step is then computed as

(14) $$R={P^{\left(\theta \right)}}^{2}W_{k}$$

The matrix $P^{\left(\theta \right)}$ is squared for analyzing similarity between rows (ranked lists) versus columns (reciprocal references). Due to the asymmetry of rank-based matrices, the multiplication by the tranposition considers similarity between ranked lists, while the reciprocal ranking references are considered without the transposition. In contrast to Equation (5), the multiplication for reciprocal analysis does not consider transposed matrices. The obtained R denotes the final result matrix which is used to define the similarity scores and the re-ranked retrieval results denoted by the set of ranked lists $\hat{T}$.

### 3.6. Rank Fusion

Several different visual features have been proposed over recent decades aiming to mimic the inherent complexity associated with the visual human perception. However, given the myriad of available features, how to combine them so that their complementarity is well exploited becomes a key question. Our answer is to derive a rank fusion approach embedded in the diffusion process.

Let $F=\{f_{0},f_{1},\cdots ,f_{m}\}$ be a set of visual features. Let $T_{i}$ be the set of ranked lists computed for a given feature $f_{i}$. The rank diffusion is computed for each feature, in order to obtain a re-ranked set ${\hat{T}}_{i}$. Based on such set of ranked lists, Equation (2) is used to derive a rank similarity matrix, given by $W_{L}\left(i\right)$, where *L* denotes the size of ranked lists and *i* stands for feature $f_{i}$. A fused similarity matrix F is defined as:

(15) $$F=\sum_{i=1}^{m}W_{L}\left(i\right)$$

Finally, the fused similarity matrix F is used to derive a novel set of ranked lists, which is submitted to the proposed rank diffusion process.

## 4. Efficiency and Complexity Aspects

In this section, we discuss and present algorithms for efficiently computing the main steps of the proposed method. Inspired by [17], the algorithm identifies high similarities values indexed through ranking information according to top-*L* positions, while discards the remaining information which results in the sparsity of the transition matrix P.

**Figure 2:**
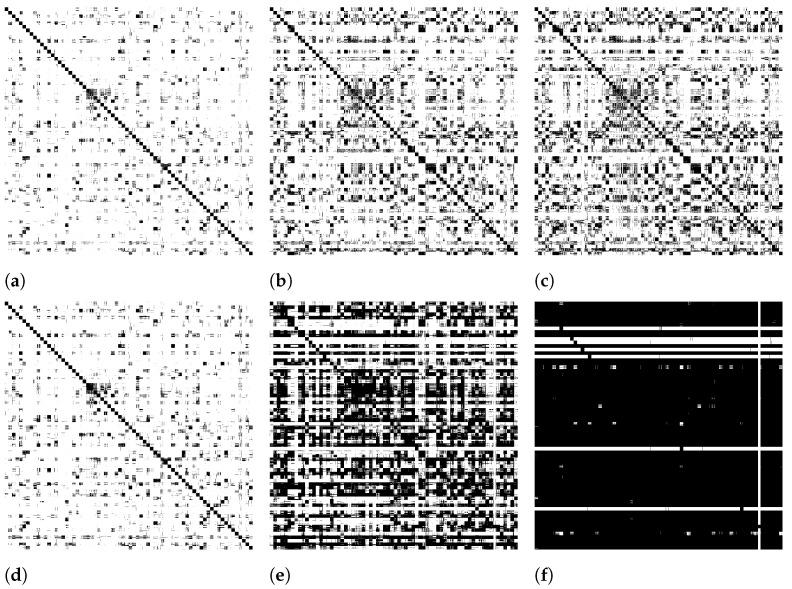
Impact of top-L constrained diffusion on sparsity of the probability matrix (first row) P on MPEG-7 dataset: non-zero values represented as black pixels. (**a**) 2nd it. L = 400, (**b**) 5th it. L = 400, (**c**) 10th it. L = 400, (**d**) 2nd it. L = Full, (**e**) 5th it. L = Full, (**f**) 10th it. L = Full.

Figure 2 illustrates the impact of our approach on the sparsity along iterations. First line depicts the matrix P with a constrained top-*L* diffusion, while the second line considers the whole dataset. In this way, the value of *L* can be seen as a trade-off parameter between effectiveness and efficiency. As experimentally evaluated in Section 5, the impact of lost information after top-*L* is not significant, even for relatively small values of *L*.

### 4.1. Efficient Algorithmic Solutions

For deriving the algorithms, we exploit a neighborhood set $N(y_{q},s)$, which contains the *s* most similar images to a given image $y_{q}$. The first main step of the method consists in the computation of the affinity matrix $W_{L}$ and its rank normalization. Algorithm 1 addresses the efficient computation of $W_{L}$ constrained to top-*L* rank positions according to Equation (2).
**Algorithm 1** Rank Sim. Measure**Require:** Set of ranked lists T, Parameter size *L***Ensure:** Sparse matrix $W_{L}$1: **for all** $y_{i}\in C$ **do**
2:  **for all** $y_{j}\in N\left(y_{i},L\right)$ **do**
3:   $w_{L_{ij}}\leftarrow p^{\tau_{i}\left(j\right)}$
4:  **end for**
5: **end for**


The efficient rank normalization is presented in Algorithm 2, which is equivalent to Equation (3). Line 2 process aims at considering most of non-zero entries for each row, which can reach 2×L. In general, presented algorithms follow the same principle of bounding the processing to the top ranking positions, which are used to discard sparse positions of matrix W and P.
**Algorithm 2** Rank Normalization**Require:** Set of ranked lists T, matrix $W_{L}$, Parameter *L***Ensure:** Reciprocal normalized set of ranked lists $\bar{T}$1: **for all** $y_{i}\in C$ **do**
2:  **for all** $y_{j}\in N\left(y_{i},2\times L\right)$ **do**
3:   ${\bar{w}}_{L_{ij}}\leftarrow w_{L_{ij}}+w_{L_{ji}}$
4:  **end for**
5: **end for**
6: $\bar{T}\leftarrow stableSorting\left(T,{\bar{W}}_{L}\right)$


The normalization procedures given by Equation (4) are addressed in Algorithm 3. The same algorithm can be used for computing the normalization of matrix P before the reciprocal analysis, by using the constant *L* instead of *k*.
**Algorithm 3** Matrix Normalization**Require:** Matrix $W_{k}$**Ensure:** Normalized Matrix $W_{k}$1: **for all** $y_{j}\in C$ **do**
2:  $a_{j}\leftarrow 0$
3: **end for**
4: **for all** $y_{i}\in C$ **do**
5:  **for all** $y_{j}\in N\left(y_{i},k\right)$ **do**
6:   $a_{j}=a_{j}+{w_{k}}_{ij}$
7:  **end for**
8: **end for**
9: **for all** $y_{i}\in C$ **do**
10:  **for all** $y_{j}\in N\left(y_{i},k\right)$ **do**
11:   $w_{ij}=w_{ij}/a_{j}$
12:  **end for**
13: **end for**


Algorithm 4 presents the proposed approach for computing the rank diffusion, defined by Equation (5). It is the central element of the proposed method, and it is iterated θ times. An analogous solution can be used to compute the post-diffusion reciprocal analysis, defined in Equation (14).
**Algorithm 4** Rank Diffusion**Require:** Matrices $W_{k}$ and $P^{t}$**Ensure:** Matrix $P^{t+1}$1: **for all** $y_{i}\in C$ **do**
2:  ${p^{\left(t+1\right)}}_{ii}\leftarrow \left(1-\alpha \right)$
3:  **for all** $y_{j}\in N\left(y_{i},L\right)$ **do**
4:   **if** $y_{i}\neq y_{j}$ **then**
5:    ${p^{\left(t+1\right)}}_{ij}\leftarrow 0$
6:   **end if**
7:   **for all** $y_{l}\in N\left(y_{j},k\right)$ **do**
8:    ${p^{\left(t+1\right)}}_{ij}\leftarrow {p^{\left(t+1\right)}}_{ij}+\left(p_{il}^{\left(t\right)}\times {w_{k}}_{jl}\right)$
9:   **end for**
10:   ${p^{\left(t+1\right)}}_{ij}\leftarrow \alpha \left({p^{\left(t+1\right)}}_{ij}\right)$
11:  **end for**
12: **end for**


### 4.2. Complexity Analysis

As discussed before, the diffusion processes typically exhibits an asymptotic complexity of $O(n^{3})$, mainly due to successive matrices multiplications required. It occurs because both relevant and non-relevant similarity information are processed. In contrast, our approach exploits rank information, which allows the algorithms presented in the previous section to compute only relevant similarity scores.

The inputs are ranked lists, which can be computed by employing an efficient k-NN graph construction method with the NN-Descent algorithm [30] or other recent approaches [31,32]. Based on the ranked lists, the sparse affinity matrix can be computed in O(n) according to Algorithm 1. Since *L* is a constant, only the loop in lines 1–5 depend on the number of elements in the dataset.

Algorithms 2 and 3 are also O(n) for analogous reasons. All the internal loops are constrained to constants *L* or *k*. The sorting step in Algorithm 2 is also performed until a constant *L*, being O(1) for each ranked list and O(n) for the whole dataset.

The most computationally expensive step is given by Algorithm 4. However, notice that loops in lines 3–11 and 7–9 are constrained to constants *L* and *k*, respectively, keeping the asymptotic complexity of O(n). This algorithm is iterated θ times, where θ is also constant. Therefore, we can conclude that all the algorithms can be computed in O(n).

### 4.3. Regional Diffusion for Unseen Queries

Let $y_{u}$ be an unseen query image, defined outside of the collection, such that $y_{u}\notin C$. In fact, such situation represents a classical and common real-world image retrieval scenario. The objective is to efficiently obtain retrieval results re-ranked by the proposed diffusion process. The main idea of our solution is computing a regional diffusion, constrained only to the top-*L* images of the unseen query ranking.

Firstly, an initial neighborhood set $N(y_{u},L)$ and a corresponding ranked list $\tau_{u}$ can be obtained through an efficient k-NN search approach [30,31,32]. The neighborhood set $N(y_{u},L)$ is used to define a sub-collection $C_{u}\subset C$, such that $|C_{u}|=L$. Next, the set of pre-computed ranked lists for images in $C_{u}$ are updated in order to contain only images of the sub-collection, removing the other images. Formally, let $y_{i},y_{j}\in C_{u}$ be two images of the sub-collection. The updated ranked list $\tau_{i}^{\prime }$ is defined as a bijection from the set $C_{u}$ onto the set [L]={1,2,⋯,L}. The position of image $y_{j}$ in the ranked list $\tau_{i}^{\prime }$ is defined as:

(16) $$\tau_{i}{\left(j\right)}^{\prime }=\left|\left\{y_{r}|y_{r}\in C_{u}\wedge \tau_{i}\left(r\right)<\tau_{i}\left(j\right)\right\}\right|+1$$

Once the ranked lists are updated, the Rank Diffusion Process is executed for the sub-collection $C_{u}$ in order to obtain the re-ranked retrieval results for the unseen query. As all the procedures are constrained to *L*, the time complexity is O(1). As discussed in experimental section, the results can be obtained in on-line time without significant effectiveness losses in comparison to the global diffusion, defined for the whole collection.

## 5. Experimental Evaluation

This section discusses the comprehensive experimental evaluation conducted in order to assess the effectiveness of the proposed method.

### 5.1. Experimental Protocol and Implementation Aspects

For image retrieval, the proposed method was evaluated on 6 diversified public datasets, ranging from 280 to 72,000 images. Different features were considered, including global (shape, color, and texture), local, mid-level representations and convolutional neural network-based features. Table 1 presents the datasets and the features used for each dataset.

All images are considered as query images, except for the Holidays [44] dataset, where we use 500 queries for comparison purposes. The effectiveness measure considered for most of experiments is the Mean Average Precision (MAP), but other measures are also considered according to the specific protocol of some datasets: the Recall at 40 (bull’s eye score) for MPEG-7 [37] dataset. For the most of experiments we also report the relative gains obtained, which is defined as follows: let $M_{b}$, $M_{a}$ be the effectiveness measures respectively before and after the use of the method, the relative gain is defined as $G=\left(M_{a}-M_{b}\right)/M_{b}$.

Regarding implementation aspects, the proposed method was developed in C++ language under the UDLF framework [61]. The framework provides a software environment to easily implement, use, and evaluate unsupervised post-processing methods. The source-code is publicly available on GitHub https://github.com/UDLF/UDLF/ (accessed on 5 March 2021), under the terms of the GPLv2 license, allowing free access and possibility of sharing the code.

### 5.2. Parametric Space Analysis

This section discusses the impact of parameters on the retrieval results. The parameters considered are: θ, *k*, α, *p*, $p_{L}$, and *L*. The number of iterations is given by θ. However, in all experiments we define θ = *k*. Therefore, *k* is the most relevant parameter since it defines both the size of neighborhood and number of iterations. The weight of identity matrix is defined by α. The probability parameter for the rank similarity measure is given by *p*, which can assume a different value $p_{L}$ during the rank normalization step. The size of ranked lists is defined by *L*.

**Figure 3:**
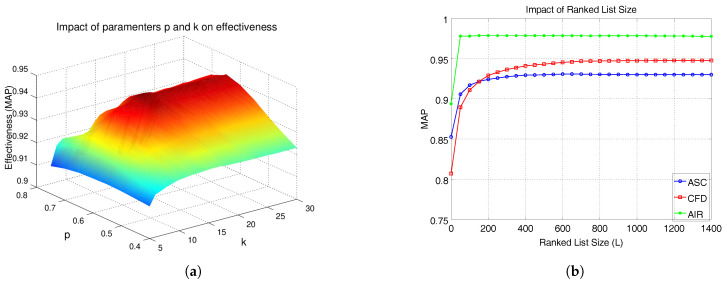
Parametric space analysis: impact of parameters *p*, *k*, and *L* on effectiveness. (**a**) Impact of parameters *p* and *k*. (**b**) Impact of ranked lists size *L*.

Experiments were conducted to analyze the impact of parameters on effectiveness. We consider the MPEG-7 with CFD as shape descriptor. Firstly, we analyzed the impact of *k* and *p* on MAP scores. Figure 3a illustrates the results. We can observe a smooth surface, which indicates the robustness of the method to different parameter settings.

The parameter *L*, which defines a trade-off between effectiveness and efficiency was also evaluated. Figure 3b presents the results for three shape descriptors: CFD, ASC, and AIR. We can observe that most of the effectiveness gains are obtained for small values of *L*. Based on the analysis, we defined the parameters (*k* = 15, α = 0.95, *p* = 0.60, $p_{L}$ = 0.99, *L* = 400), which are used for most of experiments. For the Holidays dataset, which presents a very small number of images per class, we used *k* = 4, *p* = 0.25, $p_{L}$=0.75, and *L* = 200. For the ALOI and the Person Re-ID datasets, which are larger collections, we used *L* = 1000.

### 5.3. General Image Retrieval Results

The effectiveness results obtained by our method are discussed in this section. Table 2 presents the results for shape, color, and texture features on datasets MPEG-7, Soccer, and Brodatz. The most effective results for each dataset are highlighted in bold. We can observe very significant gains, ranging from +7.17% to +35.17%.

Natural image retrieval tasks were evaluated on datasets Corel5K and Holidays. The results are presented in Table 3 and Table 4. Very impressive gains can be observed, especially on Corel5K. The best features reached a MAP score of 28.07%, while our method reached 39.45% for a single feature and 56% in a rank fusion task.

Table 5 presents the results on the ALOI dataset. Our method also achieved high-effectiveness gains, even using a small value of *L* in comparison with the size of the dataset. The retrieval results based on CNN-RESNET features were improved from 79.49% to 91.31%. We also evaluated our results for the unseen query scenarios for ALOI, which is the largest dataset considered in the experimental evaluation. Table 6 presents the MAP results for 500 randomly selected queries, one from each class. Notice that the unseen queries execution achieved results close to the full execution for all the cases.

### 5.4. Person Re-ID Results

The proposed method is also evaluated on Person Re-ID tasks. Table 7 presents information about the considered datasets, with up to 36,411 different person bounding boxes. Both are publicly available and commonly used in the literature. The MAP is reported following the protocol proposed by the dataset authors [62,63]. All the results consider the single-shot (single-query) analyzes, where only one probe image is provided per query. In the evaluation, gallery images are ranked in comparison to the probe images. Gallery images that are of the same view/cam of the probe are excluded. The training images are considered for diffusion, but their labels are not used in any of the steps.

Table 8 and Table 9 present the results for the datasets Market1501 and DukeMTMC, respectively. The CNNs (Convolutional Neural Networks) were trained on MSMT17 [64] and employed considering the pre-trained weights provided by Torchreid [65] https://kaiyangzhou.github.io/deep-person-reid/MODEL_ZOO.html (accessed on 5 March 2021). Notice that we achieved significant MAP values in all the cases, with gains up to +55.89%.

### 5.5. Visual Analysis

**Figure 4:**
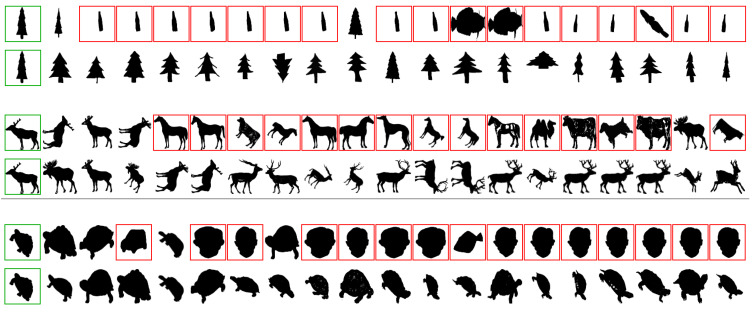
Visual retrieval results obtained on the MPEG-7 dataset.

**Figure 5:**
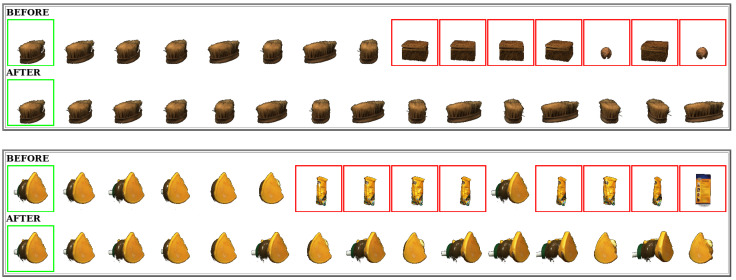
Visual retrieval results obtained on the ALOI dataset.

This section presents a visual analysis of results achieved by the proposed method. The positive impact on effectiveness is illustrated through retrieval results before and after the use of the method. Figure 4 shows the results on the MPEG-7 dataset and CFD feature considering three different queries. The effectiveness gains obtained are remarkable: the precision at top-20 positions increases from between 20% and 30% to 100% in all 3 cases. Figure 5 illustrates retrieval results for ALOI dataset. Even for a much larger dataset, very significant effectiveness gains can be observed at top ranking positions.

**Figure 6:**
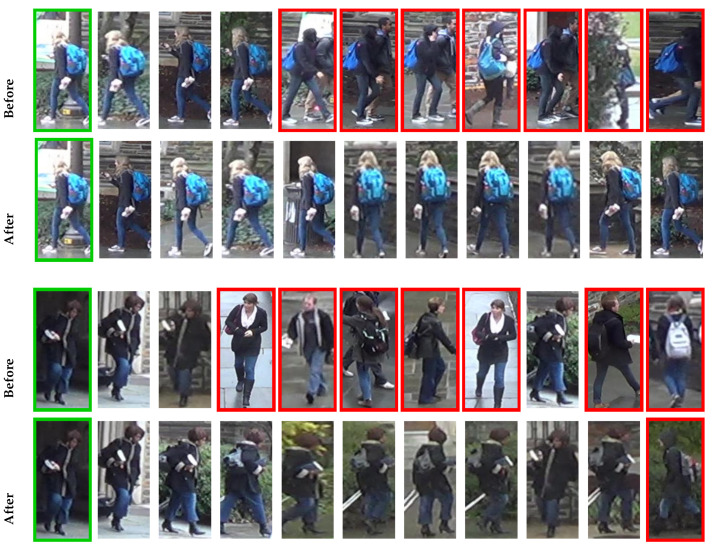
Two visual examples on DukeMTMC showing the impact of our approach.

The positive impact can also be observed on person re-ID tasks. Figure 6 shows the ranked lists for two queries on DukeMTMC dataset. The results correspond to the OSNET-AIN feature, before and after our approach was applied. The query images are presented with green borders and the incorrect results with red borders. The obtained improvements are very significant and easily noticeable.

### 5.6. Efficiency Evaluation

Section 4 presents a theoretical analysis of efficiency aspects, in which we discuss that the proposed method requires time complexity of O(n) while diffusion approaches typically have an asymptotic complexity of $O(n^{3})$. This section presents an efficiency evaluation considering the execution time of the proposed approach in comparison with other recent rank-based methods.

The comparison was performed under a common computational environment and implementation aspects. All the methods are implemented under the UDLF framework [61], using default parameter settings defined in the framework (Once default parameters were considered, it was not possible to execute RL-Sim algorithm on ALOI dataset. It is defined to use full ranked lists, such that L=n, which requires unfeasible memory amounts.). The hardware environment is composed of an Intel(R) Xeon(R) Silver 4108 CPU @ 1.80 GHz, 128 GB of memory and the software environment is given by the operating system Linux 5.8.0-44-generic-Ubuntu 20.04.1. Table 10 presents the execution time per query obtained on different datasets, considering an average of 5 executions. The efficiency results demonstrate that the proposed method is faster or comparable to the related rank-based approaches.

### 5.7. Comparison with Other Approaches

The proposed method is compared with diverse state-of-the-art related methods on two datasets commonly used as benchmark for image retrieval: MPEG-7 [37] and Holidays [44]. Table 11 reports the results on the MPEG-7 [37] in comparison with other various other post-processing methods. The bull’s eye score, which counts similar images within the top-40 rank positions, is used as effectiveness measure. Table 12 presents the MAP scores obtained on the Holidays [44] dataset, in comparison with state-of-the-art retrieval methods. On both datasets, the proposed method achieves high-effective results compared with related methods.

Our results were compared with the most recent state-of-the-art person re-ID methods. The comparison is presented in Table 13. We report the best results obtained by our method (re-ranking of OSNET-AIN). The abbreviations in parentheses indicate the datasets used for training (C02 = CUHK02, C03 = CUHK03, M = Market1501, D = DukeMTMC, MT = MSMT17). For example, the use of (D,M) indicates that it was trained on DukeMTMC (source) and tested on Market1501 (target) or the opposite. Methods that consider the target dataset labels for training are not included, since we intend to keep the comparison fair with our protocol, which is unsupervised. Notice that we achieved the highest mean among all methods, best MAP on DukeMTMC, and the second best on Market1501 (only behind ISSDA [83]).

## 6. Conclusions

In this work, we introduce a rank diffusion process for post-processing tasks in image retrieval scenarios. The proposed method embraces key advantages from both diffusion and ranked-based approaches, while avoiding most of their disadvantages. Formally defined as a diffusion process, the method is proved to converge, different from most of rank-based approaches. In addition, the method can be computed by low-complexity algorithms, in contrast to most diffusion methods. An extensive experimental evaluation demonstrates that significant effectiveness gains can be achieved on different retrieval tasks, considering various datasets and several visual features, evidencing the capacity of improving the retrieval results.

Concerning limitations, a relevant requirement of the proposed method consists of the need for computing the set of ranked lists for the images. Brute force strategies for computing the ranked lists can be unfeasible, especially for large-scale datasets. In this scenario, the use of the proposed method is limited to efficient approaches for obtaining the initial ranking results. Indexing and hashing approaches have been exploited for this objective. In our experimental evaluation, indexing approaches were used for larger datasets.

As future work, we intend to investigate if other diffusion methods for image retrieval can be efficiently computed by exploiting the proposed approach. In addition, we also intend to investigate the application of the proposed approach in other scenarios, which require efficient computation of successive multiplication matrix procedures, similar to diffusion processes.

## Figures and Tables

**Table 1:** Image datasets and features used in the experimental evaluation.

Dataset	Size	Type	General	Descriptors	Effectiv.
			Features		Measure
Soccer [33]	280	Scenes/ Color	Composed of images from 7 soccer teams, containing 40 images per class.	Border/Interior Auto Color Correlograms (ACC) [34], Pixel Classification (BIC) [35], and Global Color Histogram (GCH) [36]	MAP
MPEG-7 [37]	1400	Images: Shape	Composed of 1400 shapes divided in 70 classes. Commonly used for evaluation of post-processing methods.	Articulation-Invariant Representation (AIR) [38], Aspect Shape Context (ASC) [39], Beam Angle Statistics (BAS) [40], Contour Features Descriptor (CFD) [41], Shape Context (IDSC) [42], and Segment Saliences (SS) [43]	MAP, Recall@40
Holidays [44]	1491	Scenes	Commonly used as image retrieval benchmark, the dataset is composed of 1491 personal holiday pictures with 500 queries.	Color and Edge Directivity Descriptor Spatial Pyramid (CEED-Spy) [45,46], ACC [34], CNN-OLDFP [47], and Convolutional Neural Network by OverFeat [48] (CNN-OverFeat)	MAP
Brodatz [49]	1776	Images: Texture	A popular dataset composed of 111 different textures divided into 16 blocks.	Color Co-Occurrence Matrix (CCOM) [50], Local Activity Spectrum (LAS) [51], and Local Binary Patterns (LBP) [52]	MAP
Corel5K [53]	5000	Objects/ Scenes	Composed of 50 categories with 100 images each class, including diverse scene content.	ACC [34], ACC Spatial Pyramid (ACC-Spy) [34,46], Color and Edge Directivity Descriptor Spatial Pyramid (CEED-Spy) [45,46], CNN by framework Caffe (CNN-Caffe) [54], FCTH Spatial Pyramid (FCTH-Spy) [46,55]	MAP
ALOI [56]	72,000	Images: Objects	Images from 1000 classes of objects, with different viewpoint and illumination.	ACC [34], BIC [35], GCH [36], Color Coherence Vectors (CCV) [57], Local Color Histograms (LCH) [58], CNN-Resnet [59], and CNN-VGG [60]	MAP

**Table 2:** Retrieval results on general image retrieval tasks.

Dataset	Feature	Original	Our	Relative
		MAP	Method	Gain
Soccer	GCH	32.24%	34.55%	+7.17%
	ACC	37.23%	45.41%	+21.97%
	BIC	39.26%	46.53%	+18.52%
	BIC+ACC	-	**49.36%**	+25.73%
MPEG-7	SS	37.67%	50.92%	+35.17%
	BAS	71.52%	82.87%	+15.87%
	CFD	80.71%	94.11%	+16.60%
	IDSC	81.70%	91.09%	+11.49%
	ASC	85.28%	92.96%	+9.01%
	AIR	89.39%	97.88%	+9.50%
	CFD+ASC	-	98.84%	+10.57%
	CFD+AIR	-	**100%**	+11.87%
Brodatz	LBP	48.40%	52.19%	+7.83%
	CCOM	57.57%	66.79%	+16.02%
	LAS	75.15%	81.49%	+8.44%
	CCOM+LAS	-	**83.80%**	+11.51%

**Table 3:** Results on the Corel5K [53] dataset.

Feature	Original	Our	Relative
	MAP	Method	Gain
FCTH-Spy	27.89%	32.31%	+15.85%
JCD-Spy	29.18%	34.35%	+17.73%
ACC	27.75%	35.38%	+27.48%
ACC-Spy	29.76%	35.93%	+20.71%
CEDD-Spy	30.01%	36.07%	+20.19%
CNN-Caffe	28.07%	39.45%	+40.53%
CNN-Caffe+ACC-Spy	-	**56.00%**	+99.50%
+CEED-Spy			

**Table 4:** Results on the Holidays [44].

Feature	Original	Our	Relative
	MAP	Method	Gain
FCTH-SPy	55.42%	57.47%	+3.70%
CNN-Caffe	64.09%	71.53%	+11.61%
ACC	64.29%	69.47%	+8.04%
CNN-OverFeat	82.59%	85.74%	+3.81%
CNN-OLDFP	88.46%	89.54%	+1.22%
ACC+CNN-OLDFP+	-	90.85%	+2.70%
CNN-OverFeat		
CNN-OLDFP+		
CNN-OverFeat	-	**91.25%**	+3.15%

**Table 5:** Results on the ALOI [56] dataset.

Feature	Original	Our	Relative
	MAP	Method	Gain
ACC	43.77%	55.32%	+26.39%
BIC	71.75%	83.87%	+16.89%
CCV	47.49%	55.50%	+16.87%
GCH	50.56%	61.25%	+21.15%
LCH	58.55%	74.92%	+27.95%
CNN-RESNET	79.49%	91.31%	+14.87%
CNN-VGG	74.88%	88.53%	+18.22%

**Table 6:** Results on ALOI [56] for unseen queries.

Feature	Original	Full	UQuery
ACC	43.19%	54.81%	54.32%
BIC	73.32%	85.74%	83.30%
CCV	47.09%	55.12%	53.50%
GCH	51.16%	62.79%	61.78%
LCH	59.43%	76.35%	73.50%
CNN-RESNET	79.06%	91.73%	88.62%
CNN-VGG	73.74%	87.78%	85.17%

**Table 7:** Person Re-ID datasets considered in the experimental evaluation.

Dataset	IDs	BBox	Probe	Gallery	Train	Cam	Detector
Market1501 [62]	1501	32,668	3368	19,732	12,936	6	DPM
DukeMTMC [63]	1812	36,411	2228	17,661	16,522	8	Manual

**Table 8:** Results on the Market1501 [62] dataset.

Feature	Original	Our	Relative
	MAP	Method	Gain
MLFN [66]	21.98%	31.85%	+44.90%
HACNN [67]	23.30%	33.55%	+43.99%
RESNET [59]	22.82%	35.52%	+54.57%
OSNET [68]	37.36%	54.85%	+46.81%
OSNET-IBN [69]	37.13%	54.54%	+46.89%
OSNET-AIN [69]	43.30%	**59.82%**	+38.15%

**Table 9:** Results on the DukeMTMC [63] dataset.

Feature	Original	Our	Relative
	MAP	Method	Gain
MLFN [66]	28.98%	44.79%	+54.55%
HACNN [67]	25.57%	39.86%	+55.89%
RESNET [59]	32.00%	49.44%	+54.50%
OSNET [68]	45.20%	63.19%	+39.80%
OSNET-IBN [69]	45.52%	63.88%	+40.33%
OSNET-AIN [69]	52.69%	**67.29%**	+27.71%

**Table 10:** Execution time per query (in milliseconds) for different methods and datasets.

	Our Method	BFSTree [70]	RL-Sim [6]	LHRR [26]
MPEG-7	0.5910 ± 0.0018	0.7149 ± 0.0328	0.6888 ± 0.0817	0.6228 ± 0.0013
Corel5k	1.30356 ± 0.0009	1.3406 ± 0.0433	2.0718 ± 0.0756	2.7216 ± 0.0005
Market	7.5463 ± 0.2219	10.2885 ± 0.0536	27.6236 ± 0.4692	3.6000 ± 0.0038
ALOI	6.9376 ± 0.0272	10.8621 ± 0.6817	–	1.8177 ± 0.00049

**Table 11:** Comparison with other post-processing methods on the MPEG-7 [37] dataset.

Shape Descriptors		
CFD [41]	-	84.43%
IDSC [42]	-	85.40%
AIR [38]	-	93.67%
**Post-Processing Methods**		
**Algorithm**	**Descriptor(s)**	**Bull’s eye**
		**score**
Graph Transduction [71]	IDSC [42]	91.00%
Self-Smoothing Operator [7]	IDSC [42]	92.77%
Local Constr. Diff. Process [8]	IDSC [42]	93.32%
Shortest Path Propagation [72]	IDSC [42]	93.35%
**Our Method**	**IDSC [42]**	**93.40%**
SCA [11]	IDSC [42]	93.44%
Correlation Graph [73]	CFD [41]	94.27%
RL-Sim [6]	CFD [41]	94.27%
Rank Diffusion [17]	CFD [41]	96.19%
Reciprocal kNN Graph + CCs [12]	CFD [41]	96.61%
**Our Method**	**CFD [41]**	**96.74%**
RL-Sim [6]	AIR [38]	99.94%
Tensor Product Graph [4]	AIR [38]	99.99%
Generic Diffusion Process [5]	AIR [38]	100%
Neighbor Set Similarity [23]	AIR [38]	100%
**Our Method**	**AIR [38]**	**100%**

**Table 12:** Comparison with retrieval approaches on the Holidays [44] dataset.

MAP Scores for State-of-the-Art Methods				
Tolias	Paulin	Qin	Zheng	Sun
et al. [74]	et al. [75]	et al. [76]	et al. [77]	et al. [78]
82.20%	82.90%	84.40%	85.20%	85.50%
Zheng	Pedronette	Iscen	Li	Liu
et al. [79]	et al. [12]	et al. [80]	et al. [81]	et al. [82]
85.80%	86.19%	87.5%	89.20%	90.89 %

**Our Method:** 91.25%.

**Table 13:** Comparison with state-of-the-art Person Re-ID methods-MAP (%).

	Person re-ID Datasets		
	**Market1501**	**DukeMTMC**	**Mean**
**Unsupervised Methods**			
ARN [84]	39.4	33.4	36.4
EANet [85]	40.6	26.4	33.5
ECN [86]	43.0	40.4	41.7
MAR [87]	40.0	48.0	44.0
TAUDL [88]	41.2	43.5	42.4
UTAL [89]	46.2	44.6	45.4
**Domain Adaptive Methods**			
HHL (D,M) [90]	31.4	27.2	26.6
HHL (C03) [90]	29.8	23.4	25.0
ATNet (D,M) [91]	25.6	24.9	25.3
CSGLP (D,M) [92]	33.9	36.0	35.0
ISSDA (D,M) [83]	**63.1**	54.1	58.6
**Cross-Domain Methods**			
EANet (C03) [85]	33.3	22.0	27.7
EANet (D,M) [85]	32.9	31.7	32.3
SPGAN (D,M) [93]	17.0	16.7	16.9
DAAM (D,M) [94]	17.5	14.5	16.0
AF3 (D,M) [95]	36.3	37.4	36.9
AF3 (MT) [95]	37.7	46.2	42.0
PAUL (MT) [96]	40.1	53.2	46.7
EMTL (C02+D+M) [97]	25.1	22.3	23.7
CAMEL [98]	26.3	—	26.3
Baseline by [99]	56.8	46.9	51.9
**Proposed Approach**			
**Our Method**	**59.82**	**67.29**	**63.56**

## Data Availability

Public datasets and code used are properly referenced along with the paper.
